# Evaluation of deep‐learning image reconstruction for chest CT examinations at two different dose levels

**DOI:** 10.1002/acm2.13871

**Published:** 2022-12-30

**Authors:** Angelica Svalkvist, Erika Fagman, Jenny Vikgren, Sara Ku, Micael Oliveira Diniz, Rauni Rossi Norrlund, Åse A. Johnsson

**Affiliations:** ^1^ Department of Medical Physics and Biomedical Engineering Sahlgrenska University Hospital Gothenburg Sweden; ^2^ Department of Medical Radiation Sciences Institute of Clinical Sciences The Sahlgrenska Academy at University of Gothenburg Gothenburg Sweden; ^3^ Department of Radiology Sahlgrenska University Hospital Gothenburg Sweden; ^4^ Department of Radiology Institute of Clinical Sciences Sahlgrenska Academy University of Gothenburg Gothenburg Sweden

**Keywords:** chest, computed tomography, deep‐learning image reconstruction, post‐processing, ultra‐low dose

## Abstract

**Aims:**

The aims of the present study were to, for both a full‐dose protocol and an ultra‐low dose (ULD) protocol, compare the image quality of chest CT examinations reconstructed using TrueFidelity (Standard kernel) with corresponding examinations reconstructed using ASIR‐V (Lung kernel) and to evaluate if post‐processing using an edge‐enhancement filter affects the noise level, spatial resolution and subjective image quality of clinical images reconstructed using TrueFidelity.

**Methods:**

A total of 25 patients were examined with both a full‐dose protocol and an ULD protocol using a GE Revolution APEX CT system (GE Healthcare, Milwaukee, USA). Three different reconstructions were included in the study: ASIR‐V 40%, DLIR‐H, and DLIR‐H with additional post‐processing using an edge‐enhancement filter (DLIR‐H + E2). Five observers assessed image quality in two separate visual grading characteristics (VGC) studies. The results from the studies were statistically analyzed using VGC Analyzer. Quantitative evaluations were based on determination of two‐dimensional power spectrum (PS), contrast‐to‐noise ratio (CNR), and spatial resolution in the reconstructed patient images.

**Results:**

For both protocols, examinations reconstructed using TrueFidelity were statistically rated equal to or significantly higher than examinations reconstructed using ASIR‐V 40%, but the ULD protocol benefitted more from TrueFidelity. In general, no differences in observer ratings were found between DLIR‐H and DLIR‐H + E2. For the three investigated image reconstruction methods, ASIR‐V 40% showed highest noise and spatial resolution and DLIR‐H the lowest, while the CNR was highest in DLIR‐H and lowest in ASIR‐V 40%.

**Conclusion:**

The use of TrueFidelity for image reconstruction resulted in higher ratings on subjective image quality than ASIR‐V 40%. The benefit of using TrueFidelity was larger for the ULD protocol than for the full‐dose protocol. Post‐processing of the TrueFidelity images using an edge‐enhancement filter resulted in higher image noise and spatial resolution but did not affect the subjective image quality.

## 1 INTRODUCTION

Ever since the first introduction of computed tomography (CT) in the 1970s, a steady technical development has been ongoing. Due to wide detector arrays and fast gantry rotation times, modern CT scanners can perform whole‐body examinations in only a couple of seconds.^1^ The relatively short examinations times combined with the large amount of obtained diagnostic information has made CT a valuable tool in the clinical patient care. Today CT is frequently used both for diagnosis of disease and for follow‐up of treatment and the number of annually performed CT examinations continue to rise. Due to the change in ICRP tissue weighting factors in 2007 it is however somewhat difficult to compare average effective doses from CT examinations over the years. In a study aiming at investigating diagnostic imaging trends in the United States^2^ it was shown that the annual effective dose per capita from CT examinations was 0.38 mSv in 1996 (calculated using the tissue weighting factors of ICRP 103^3^). This number had increased to 1.58 mSv per capita in 2010. Mettler et al.^4^ have summarized the content in NCRP Report 184 on medical radiation exposure of patients in the United States^5^ with focus on the trends regarding number of CT procedures and resulting average patient effective doses from 2006 to 2016. It is shown that the number of performed CT procedures increased from a total of 62 million in 2006 to a total of 74 million in 2016. The corresponding average individual effective doses were 1.46 mSv in 2006 and 1.45 mSv in 2016, calculated using the tissue weighing factors from ICRP Publication 60.^6^ The average individual effective dose was 1.37 mSv in 2016 when calculated using the tissue weighting factors from ICRP publication 103. These results indicate that the technical developments during the last decade aiming at reducing the radiation dose from CT examinations^1^ might have had desired effect. There is however a relatively large variation in radiation dose between different countries. For example, an analysis of the variation in mean effective doses from CT examinations between seven different countries revealed that the effective dose from chest CT examinations varied with almost a factor of 4 (from 1.7 mSv in Switzerland to 6.4 mSv in the United States).^7^

Even though future technical developments might increase the possibilities to lower the radiation doses from CT examinations even further, there will always be a relationship between radiation dose and image quality. In general, image quality can always be improved by increasing the radiation dose. Therefore, the “as low as reasonable achievable” (ALARA) principle^8^ is crucial in the optimization of CT examinations. One of the key parameters affecting the final image quality in CT is the reconstruction algorithm used. The desire to be able to perform CT examinations at lower dose levels without compromising image quality has therefore led to an extensive development of new reconstruction algorithms during the past decade.^9^, ^10^ Before the 2010s filtered back projection (FBP) was the method commonly used for reconstruction in CT. In 2009 the first iterative‐based reconstruction technique became clinically available. Compared to FBP, iterative reconstruction (IR) has been shown to decrease the image noise. However, IR have also been shown to alter the noise properties in the images resulting in “plastic‐looking” images.^11^ Because of this unfamiliar image appearance many radiologists might hesitate to fully implement IR in the clinical routine. In an attempt to overcome this identified drawback, GE Healthcare recently presented a new, deep learning‐based image reconstruction (DLIR) algorithm, commercially named TrueFidelity (GE Healthcare, Wisconsin, USA).

Previous phantom studies have shown that TrueFidelity produces images with reduced noise and higher spatial resolution than both FBP and adaptive statistical IR (ASIR‐V, GE Healthcare, Wisconsin, USA), while keeping the same noise texture as FBP.^12^, ^13^, ^14^, ^15^, ^16^ The clinical benefits of TrueFidelity over ASIR‐V and FBP in chest CT have also been demonstrated in different patient studies. For example, in a study by Hata et al.^17^ both objective and subjective image quality was evaluated in contrast‐enhanced chest CT examinations. It was shown that medium and high levels of TrueFidelity (DLIR‐M and DLIR‐H) produced reconstructed CT images with higher signal‐to‐noise ratio (SNR) and higher contrast‐to‐noise ratio (CNR) than ASIR‐V 60%, as well as higher scores on the subjective image quality assessment regarding presence of noise, streak artefacts and overall image quality. In a different study, Kim et al.^18^ evaluated image quality and noise in CT images reconstructed using TrueFidelity. The evaluations were based on low‐dose chest CT examinations (mean effective dose 0.75 mSv) and the results revealed that TrueFidelity (DLIR‐M and DLIR‐H) produced images with significantly higher SNR and CNR than ASIR‐V 30%. The images reconstructed using TrueFidelity also obtained a significantly higher score than ASIR‐V 30% on subjective image quality assessment regarding noise and contrast.

Today TrueFidelity is only available using the Standard kernel. Therefore, previous comparisons between the two reconstruction techniques are based on the use of the Standard kernel for image reconstruction.^12^, ^13^, ^14^, ^16^, ^17^, ^18^, ^19^ In the clinic, both a hard (edge preserving) convolution kernel and a soft (edge smoothing) convolution kernel are commonly used as compliments to each other for evaluation of chest CT examinations.^20^ The Lung kernel is a sharper kernel than the Standard kernel which means that the Lung kernel produces images with higher spatial resolution and increased image noise compared to the Standard kernel.^21^, ^22^ Hence, even though TrueFidelity seem to produce images with reduced noise and higher spatial resolution than ASIR‐V using the Standard kernel, the comparison between TrueFidelity and ASIR‐V might result in a slightly different outcome if the Lung kernel is used for the ASIR‐V reconstruction in the comparison. If it is shown that TrueFidelity with Standard kernel also produces images of equal or higher image quality than ASIR‐V with Lung kernel, there might be an opportunity to replace two reconstructed series with only one reconstructed image series in the clinical evaluation of chest CT examinations. In previous studies it has however been shown that for examinations performed using CT Dose Index Volume (CTDI_vol_) values ≤1 mGy the high‐contrast spatial resolution is actually higher in images reconstructed using ASIR‐V than in images reconstructed using TrueFidelity.^12^, ^13^, ^14^, ^15^ This indicates that there might be a loss in spatial resolution when TrueFidelity is used for image reconstruction of examinations performed at very low dose levels. This loss in spatial resolution might be reduced if the images reconstructed using TrueFildelity are post‐processed using an edge‐enhancement filter. On the other hand, such a post‐processing might lead to an increase in noise level in the reconstructed images. Therefore, the aims of the present study were to, for both a full‐dose protocol and an ultra‐low dose (ULD protocol, compare the image quality of chest CT examinations reconstructed using TrueFidelity (Standard kernel) with corresponding examinations reconstructed using ASIR‐V with the Lung kernel and to evaluate if post‐processing using an edge‐enhancement filter affects the noise level and subjective image quality of clinical images reconstructed using TrueFidelity.

## 2 METHODS

### 2.1 Study population

Between April and June of 2021, a total of 25 consecutive patients, referred to the radiological department at Sahlgrenska University Hospital for a thoracic CT without intra‐venous contrast were included in the study. Information regarding patient gender, age, weight, and height at the time of the examination were recorded.

### 2.2 Deep learning image reconstruction

According to a technical white paper,^23^ the DLIR commercially named TrueFidelity is based on the use of a deep neural network (DNN) that is created by multiple layers of mathematical equations. These equations can be used to manipulate the input data in order obtain a desired output. In order to accomplish this task, the DNN was trained using a large number of examinations pairs, consisting of a high‐dose scan and a low‐dose scan of the same object. The TrueFidelity reconstruction was applied to the low‐dose scan and produced reconstructed images as an output. The output images were then compared to the true high‐dose images (reconstructed using FBP) and based on this comparison the mathematical functions in the DNN were adjusted in order to minimize the difference in image features between the TrueFidelity output and the known truth. After training, the DLIR engine was tested using large amounts different data sets to ensure the accuracy of the produced DLIR engine. When performing reconstructions using TrueFidelity, three different reconstruction strength levels are available: DLIR‐low (DLIR‐L), DLIR‐medium (DLIR‐M), and DLIR‐high (DLIR‐H). The different levels correspond to different levels of noise reduction in the reconstructed images (from low to high). As mentioned previously, at the time of the study TrueFidelity is only available using the standard kernel.

### 2.3 CT image acquisition and reconstruction

The CT examinations were performed using a GE Revolution APEX CT (GE Healthcare, Milwaukee, USA). After two initial scout images, the patients were examined using a spiral CT series with a clinical full‐dose protocol. For study purposes all included patients also underwent an additional ULD spiral series. The exposure level of the ULD protocol were chosen to result in an effective dose to a standard‐sized patient of approximately 0.05 mSv, which is the same dose level as the effective dose to a standard‐sized patient from a conventional chest x‐ray examination (frontal + lateral image) at the hospital.^24^ A summary of scan parameters for the two protocols are shown in Table 1. For each performed examination using the two protocols, information regarding CTDI_vol_ and dose length product (DLP) were recorded. The resulting effective dose for each protocol was estimated using a conversion factor between DLP and effective dose for chest CT (0.015 mSv/mGy·cm^25^).

**TABLE 1:** Scan parameters used for the chest CT examinations performed using the full‐dose protocol and the ultra‐low (ULD) protocol

Parameter	Full‐dose protocol	ULD protocol
Scan type	Helical	Helical
Scan field‐of‐view	Large body	Large body
Collimation	80 mm	80 mm
Rotation time (s)	0.5	0.28
Pitch	0.992	1.531
Slice thickness (mm)	0.625	1.25
Noise Index	31	85
Kilovoltage (kV)	120	100
Milliampere (mA) range	80–560	10–15
ASIR‐V level	40%	100%
Organ dose modulation	Yes	No

During a Delphi process, three experienced thoracic radiologists (E.F., J.V., and Å.J.) reviewed patient images reconstructed using both the clinically used reconstruction ASIR‐V 40% and different settings of TrueFidelity. After completion of the Delphi process three different reconstructions were selected to be included in the study: ASIR‐V 40% (Lung kernel), DLIR‐H (Standard kernel), and DLIR‐H + E2 (Standard kernel), where E2 is a post‐processing edge‐enhancement filter (GE Healthcare, Milwaukee, USA). Data collected using both the full‐dose protocol and the ULD protocol were reconstructed using the three selected reconstruction settings, all with a reconstructed slice thickness of 0.625 mm.

### 2.4 Subjective image quality evaluation

The images were evaluated by visual assessment of specific image quality questions based on both the reproduction of different anatomical structures in the images and general image quality. A summary of the questions used for evaluation and the corresponding answering alternatives are shown in Table 2. Most of the image quality questions used in the present study (Q1–5 in Table 2) were based on the image quality criteria for chest CT examinations, established during a Delphi process including a group of experts from five different institutions participating in work package 2 of the European research project MEDIRAD ‐ Implications of Medical Low Dose Radiation Exposure, which received funding from the Euratom research and training program 2014–2018 under grant agreement No 755523.^26^ An additional criterion regarding visual reproduction of lymph node 4R (Q6 in Table 2) was added to the image evaluation in the present study. The fulfilment of each criterion was graded on a five‐step grading scale reaching from “Confident that the criterion is fulfilled” to “Confident that the criterion is not fulfilled”. In addition, the image quality evaluation in the present study included questions regarding the acceptability of the general image quality for diagnosis of four common medical conditions. The acceptability was graded on a three‐step grading scale; “fully acceptable”, “acceptable”, and “unacceptable”.

**TABLE 2:** The image quality questions used for image evaluation

Image quality questions	
Reproduction of anatomical structures	Answer alternatives
Q1. Clear reproduction of the major fissure of the left lung (right if left one is not visible)	1. Confident that the criterion is fulfilled 2. Somewhat confident that the criterion is fulfilled 3. I do not know if the criterion is fulfilled or not 4. Somewhat confident that the criterion is not fulfilled 5. Confident that the criterion is not fulfilled
Q2. Clear reproduction of B1: 3 subdivisions on axial plane of the apical bronchus of the right upper lobe (left upper lobe if right one is not visible)	
Q3. Clear reproduction of A6: 4 divisions on axial plane of right apical pulmonary artery of the right lower lobe (left lower lobe if right one is not visible)	
Q4. Clear reproduction of B6: 3 divisions on axial plane of the apical bronchus of the right lower lobe (left lower lobe if right one is not visible)	
Q5. Clear reproduction of the right inferior pulmonary vein (RIPV): 3 divisions on axial plane (left if right one is not visible)	
Q6. Clear reproduction of lymph node 4R (lymph node between VCS and carina)	

General image quality	Answer alternatives
Q7. Image quality acceptable for diagnosis of pulmonary nodules?	A. Fully acceptable B. Probably acceptable C. Unacceptable
Q8. Image quality acceptable for diagnosis of fibrosis?	
Q9. Image quality acceptable for diagnosis of emphysema?	
Q10. Image quality acceptable for diagnosis of mediastinal inflammation (fat stranding)?	

*Note*: The evaluation of questions 1–6 was made using a five‐step rating scale, while the evaluation of questions 7–10 was made using a three‐step rating scale.

Two separate observer studies were conducted, one including the examinations performed using the ULD protocol and one including the examinations performed using the full‐dose protocol. Five observers participated in the study, four thoracic radiologists with more than 10 years of clinical experience and one resident in radiology. The studies were conducted using ViewDEX 3.0,^27^ a software tool specially developed with the purpose of facilitating observer performance studies. The images were evaluated on a DICOM calibrated high‐quality medical display monitor (Eizo, Radi Force RX320), placed in a room with low ambient light. By using ViewDEX, all image series within each study were displayed to the observers in a randomized order, individual for each observer. During image evaluation the observers were allowed to adjust window width and window level, as well as to zoom and pan the images. The observers were instructed to finish the review of the study including the examinations performed using the ULD protocol before starting the review of the study including the examinations performed using the full‐dose protocol.

### 2.5 Statistical analysis of subjective image quality

The results from the observer studies were statistically analyzed using visual grading characteristics (VGC) analysis, which is a non‐parametric, rank‐invariant method for comparing visual grading data from two different imaging conditions.^28^ For each study, the ratings for one imaging condition (test condition) were plotted against the ratings for another imaging condition (reference condition) to create a VGC curve. The separation in ratings between the two imaging conditions were determined by the area under the VGC curve (AUC_VGC_). An AUC_VGC_ < 0.5 indicates higher ratings for the reference condition, while an AUC_VGC_ > 0.5 indicates higher ratings for the test condition. An AUC_VGC_ equal to 0.5 indicates similar ratings for the two conditions. The software VGC Analyzer^29^ was used to determine the AUC_VGC_ and to perform statistical analysis of the VGC data. VGC Analyzer determines the AUC_VGC_ using both the trapezoidal rule and the binormal curve fitting method. The statistical analysis of the data handles both paired and non‐paired data and is performed both for the random reader situation and for the fixed reader situation. The uncertainty of the AUC_VGC_ is determined by resampling the data using bootstrapping. In the fixed reader analysis, data from all the original observers are included when the data sets are resampled, and the results are thereby only applicable to the actual observers participating in the study. In the random reader analysis, a bootstrapping of observers is applied when the data sets are resampled, and the results are applicable to a general population of observers.

### 2.6 Quantitative evaluations of image quality

To analyze the frequency components of the images the power spectrum (PS) was determined using the same method as used for determination of the noise PS.^30^ As the analysis is based on clinical images including anatomy and not only noise, the term PS is used instead of noise PS here. Data from the reconstructed images from all of the patients included in the study was used. For each patient, imaging protocol and reconstruction method, the axial image slices from the examinations were used to extract data. The two‐dimensional PS of each image slice was determined by averaging the PS of non‐overlapping regions of interest (ROIs) of size 64 × 64 pixels placed within the central 128 × 128 pixels of each image slice. In order to reduce the edge artefacts that may occur when the PS is calculated in an image where there is a strong correlation between pixels, each small ROI was mirrored before calculation of the PS.^31^ The two‐dimensional PS of each examination was obtained by averaging the two‐dimensional PS from each image slice included in the examination. Finally, the one‐dimensional PS was obtained by radially averaging the two‐dimensional PS of each examination. To estimate the variation in PS between different patients, the relative standard error for each data point was calculated as a measure of uncertainty.

**FIGURE 1:**
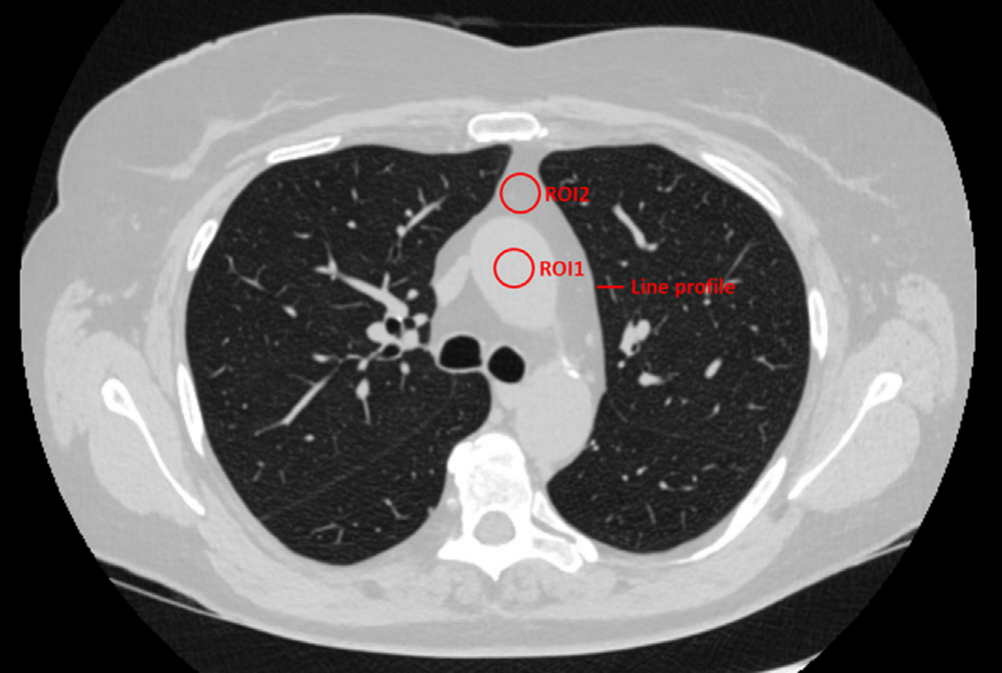
Illustration of the placement of the two ROIs (ROI1 and ROI2) used for calculation of the CNR, together with an illustration of a line profile placed perpendicular over a small vessel to evaluate spatial resolution

The reconstructed images from the patients included in the study were also used to estimate the effect of the different reconstructions on CNR and spatial resolution. Two circular ROIs (diameter 15 mm) were placed in two different locations in one of the image slices from each patient. The image slice at the level of carina was chosen and the first ROI (ROI1) was placed within the central part of aorta ascendens. The second ROI (ROI2) was placed in the mediastinal fat anterior to the aorta ascendens. In Figure 1 an example of ROI placement is illustrated. The CNR was calculated by:

(1) $$CNR\;=\frac{HU_{ROI1}-HU_{ROI2}}{\sqrt{SD_{ROI1}^{2}+SD_{ROI2}^{2}}}\;,$$

where *HU_ROI1_* is the mean Houndsfield unit (HU) value in the first ROI, *HU_ROI2_* is the mean *HU* value in the second ROI, *SD_ROI1_* is the standard deviation in the first ROI and *SD_ROI2_* is the standard deviation in the second ROI. For each dose level and patient, the relative differences in CNR between the reconstructions were calculated as CNR_Recon1_ – CNR_Recon2_, where Recon1 and Recon2 are the two reconstructions compared. The mean values of the differences, and the standard errors of the mean, were then calculated using the relative differences from all 25 patients. Student‐paired sample *t*‐test was used for statistical analysis of the relative differences. A *p*‐value < 0.05 was considered as statistically significant.

As the reconstructed images are both non‐stationary and non‐linear the spatial resolution in the images will vary between objects of different contrast. It is therefore challenging to perform a thorough quantitative evaluation of spatial resolution in anatomical images. However, to exemplify the effect of the different reconstructions on spatial resolution, line profiles were plotted perpendicular over small vessels present in the reconstructed image slices of the patients. The same coordinates for the line profile were used for all three evaluated reconstructions. In Figure 1 an example of the placement of a line profile is illustrated.

## 3 RESULTS

A summary of the patient demographics and resulting effective doses from the full‐dose protocol and the ULD protocol for the 10 male and 15 female patients included in the study are shown in Table 3. The effective dose from the ULD protocol was on average 0.05 mSv which is 2% of the mean effective dose from the full‐dose protocol (2.5 mSv).

**TABLE 3:** Patient demographics and radiation doses

Patient demographics	Mean (range)
Age (years)	66 (44–84)
Weight (kg)	77 (46–115)
Height (cm)	170 (151–188)
BMI (kg/m2)	26 (16–34)

Radiation dose full‐dose protocol	Mean (range)
CTDIvol (mGy)	4.5 (2.9–11.2)
DLP (mGycm)	169 (110–434)
Effective dose (mSv)	2.5 (1.6–6.3)

Radiation dose ULD protocol	Mean (range)
CTDIvol (mGy)	0.08 (0.07–0.1)
DLP (mGycm)	3.2 (2.7–4.2)
Effective dose (mSv)	0.05 (0.04–0.06)

**FIGURE 2:**
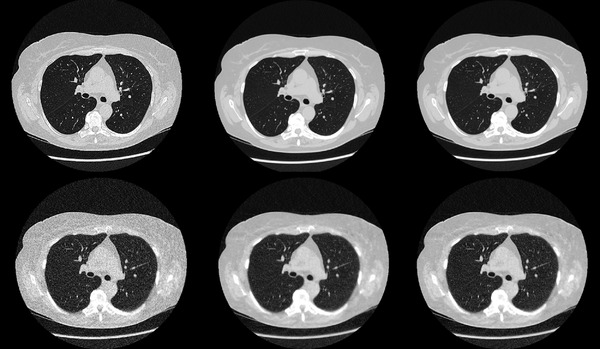
Axial CT slices obtained from the examinations performed using the full‐dose protocol (top row) and the ultra‐low dose (ULD) protocol (bottom row). The images are reconstructed using different reconstruction settings. Left: ASIR‐V 40%. Middle: DLIR‐H. Right: DLIR‐H + E2

In Figure 2 examples of CT images collected using both the full‐dose protocol and the ULD protocol and reconstructed with the evaluated reconstruction settings are shown. It is clearly seen that the noise level is higher in the images collected using the ULD protocol than in the images collected using the full‐dose protocol. Also, the level of image noise seems to be lower in the images reconstructed using TrueFidelity (DLIR‐H and DLIR‐H + E2) than in the corresponding images reconstructed using ASIR‐V 40%.

### 3.1 Visual grading characteristics studies

**FIGURE 3:**
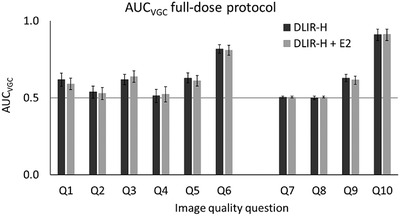
The resulting AUC_VGC_, with corresponding 95% confidence interval, resulting from the statistical analysis of the VGC rating data for examinations performed using the full‐dose protocol, where DLIR‐H and DLIR‐H + E2 is compared to the reference ASIR‐V 40%. A statistically significant difference is obtained when the AUC_VGC_ is ≠ 0.5. No statistically significant differences were found between DLIR‐H and DLIR‐H + E2

**FIGURE 4:**
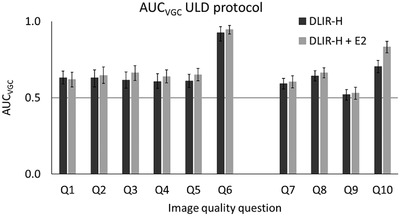
The resulting AUC_VGC_, with corresponding 95% confidence interval, resulting from the statistical analysis of the VGC rating data for examinations performed using the ultra‐low dose (ULD) protocol, where DLIR‐H and DLIR‐H + E2 is compared to the reference ASIR‐V 40%. A statistically significant difference is obtained when the AUC_VGC_ is ≠ 0.5. The only statistically significant difference between DLIR‐H and DLIR‐H + E2 was found for Q10

Figures 3 and 4 show the resulting AUC_VGC_ (including 95% CI) from the statistical analysis of the rating data from the VGC studies both for the image quality questions regarding reproduction of anatomical structures (Q1–6) and for the questions regarding general image quality (Q7–10). Due to the relatively small number of observers (*n* = 5) the results from the analysis were based on the fixed reader situation using the trapezoidal rule for curve fitting. In the comparison between TrueFidelity and ASIR‐V 40%, the latter was used as the reference condition. In the comparison between DLIR‐H and DLIR‐H + E2, DLIR‐H was used as the reference condition. Both for the full‐dose protocol and for the ULD protocol, examinations reconstructed using TrueFidelity (DLIR‐H or DLIR‐H + E2) were statistically rated significantly higher than (AUC > 0.5) or equal to (AUC = 0.5) examinations reconstructed using ASIR‐V 40% for all image quality questions. The benefit of using TrueFidelity instead of ASIR‐V 40% for image reconstruction seems to be higher for the ULD protocol than for the full‐dose protocol. For the ULD protocol, examinations reconstructed using TrueFidelity were statistically rated significantly higher than examinations reconstructed using ASIR‐V 40% for all but one of the image quality questions (Q9, image quality acceptable for emphysema?). In general, no statistically significant difference in ratings can be seen between examinations reconstructed using DLIR‐H and examinations reconstructed using DLIR‐H + E2. The only exception is found for Q10 (image quality acceptable for diagnosis of mediastinal inflammation [fat stranding]?) for the ULD protocol, where DLIR‐H + E2 statistically is rated significantly higher than DLIR‐H.

### 3.2 Detailed analysis of general image quality

**FIGURE 5:**
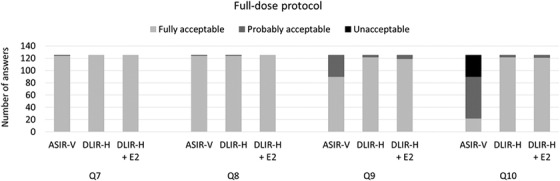
The distribution between the different answer alternatives (fully acceptable, probably acceptable, and unacceptable) in the evaluation of the general image quality questions (questions 7–10) for the examinations performed using the full‐dose protocol

**FIGURE 6:**
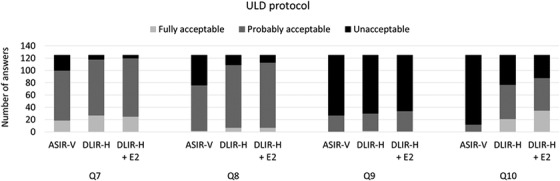
The distribution between the different answer alternatives (fully acceptable, probably acceptable, and unacceptable) in the evaluation of the general image quality questions (questions 7–10) for the examinations performed using the ultra‐low dose (ULD) protocol

The results of the VGC analysis show the ratings of the examinations reconstructed using TrueFidelity compared to images reconstructed using ASIR‐V 40%. However, these results do not show whether the general image quality for diagnosis of different conditions were rated to be acceptable or not. In Figures 5 and 6, the proportions of answers for the different answer alternatives (fully acceptable, probably acceptable, and unacceptable) are plotted for the different image quality questions regarding general image quality (Q7–10). For all general image quality questions, the proportion of examinations that were rated to have an image quality that were fully acceptable or probably acceptable were larger for examinations reconstructed using TrueFidelity (DLIR‐H or DLIR‐H + E2) than for examinations reconstructed using ASIR‐V 40%. For the full‐dose protocol (Figure 5), the image quality was rated to be fully acceptable or probably acceptable for Q7–9, regardless of reconstruction method. For diagnosis of mediastinal inflammation (fat stranding) (Q10) a proportion of examinations reconstructed using ASIR‐V 40% were rated to be of unacceptable quality, while both DLIR‐H and DLIR‐H + E2 resulted in high proportions of examinations with fully acceptable image quality and none with unacceptable image quality. For the ULD protocol (Figure 6), the image quality was, for a relatively large proportion of examinations, rated to be fully acceptable or probably acceptable for diagnosis of pulmonary nodules. In opposite, the image quality was rated to be unacceptable for diagnosis of emphysema (Q9) for most examinations, regardless of reconstruction method. For this image quality question, the use of DLIR did not have a large impact on image quality. For the ULD protocol, the largest improvement of image quality when reconstructing the examinations using DLIR or DLIR‐H + E2 instead of ASIR‐V 40% was found for Q10.

### 3.3 Quantitative analysis of image quality

**FIGURE 7:**
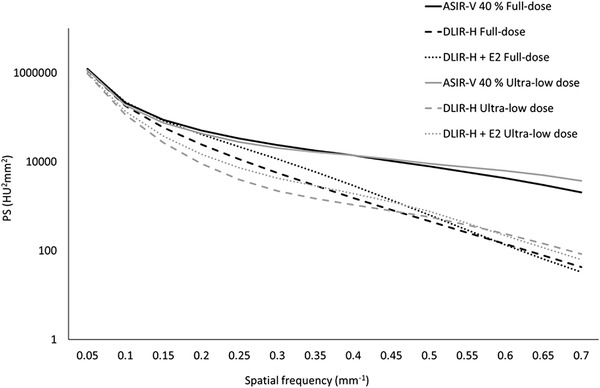
The power spectrum (PS) obtained for the different reconstructions evaluated in the present study using both the full‐dose protocol (black) and the ultra‐low dose (ULD) protocol (grey). The solid lines represent the PS for the images reconstructed using ASIR‐V 40%, the dashed lines represent the PS for images reconstructed using DLIR‐H and the dotted lines represent the PS for images reconstructed using DLIR‐H + E2. For all reconstructions and frequencies, the relative standard error of each data point is approximately 6% for the images collected using the full‐dose protocol and approximately 8% for the images collected using the ULD protocol

In Figure 7 the PS for the different reconstruction methods, averaged over all patients included in the study, are shown for both the full‐dose protocol and the ULD protocol. The PS shows that the overall pixel variance is reduced in the examinations reconstructed using TrueFidelity (DLIR‐H and DLIR‐H + E2) compared to ASIR‐V 40% for both protocols. For TrueFidelity, the overall pixel variance is slightly increased when the edge‐enhancement filter E2 is added to the reconstructed images (DLIR‐H + E2). This increase in overall pixel variance did however not result in lower image quality ratings (see Figures 3 and 4). The same behavior of the PS for the different reconstructions is found also when the PS of each patient is analyzed individually. In this study, quantum noise as commonly reported in x‐ray imaging was not measured due to the presence of underlying anatomy. It can however be reasonably assumed that the same anatomical variations were included in the measurement of PS for each of the six reconstructions for each patient (three recons × two dose levels), indicating that the higher overall pixel variance in the ASIR‐V reconstructions is due to a higher amount of quantum noise.

In Table 4 the relative differences in CNR between ASIR‐V 40%, DLIR‐H and DLIR‐H + E2 are shown. All relative differences are statistically significant (*p*‐value < 0.05). For both the full‐dose protocol and the ULD protocol, the CNR is increased when DLIR‐H or DLIR‐H + E2 is used for image reconstruction instead of ASIR‐V 40%. The CNR is slightly higher in the images reconstructed using DLIR‐H than in the images reconstructed using DLIR‐H + E2.

**TABLE 4:** The mean relative difference in contrast‐to‐noise ratio (CNR) and the standard error of the mean (SEM) for the different reconstruction settings

	Full‐dose protocol		
	Mean relative difference in CNR	SEM	*p*‐value
DLIR‐H vs. ASIR‐V 40%	7.5	0.4	<0.05
DLIR‐H + E2 vs. ASIR‐V 40%	5.8	0.3	<0.05
DLIR‐H vs. DLIR‐H + E2	1.7	0.1	<0.05
	**ULD protocol**		
	**Mean relative difference in CNR**	**SEM**	***p*‐value**
DLIR‐H vs. ASIR‐V 40%	3.0	0.1	<0.05
DLIR‐H + E2 vs. ASIR‐V 40%	2.3	0.1	<0.05
DLIR‐H vs. DLIR‐H + E2	0.6	0.04	<0.05

*Note*: A *p*‐value < 0.05 indicates statistically significant difference.

**FIGURE 8:**
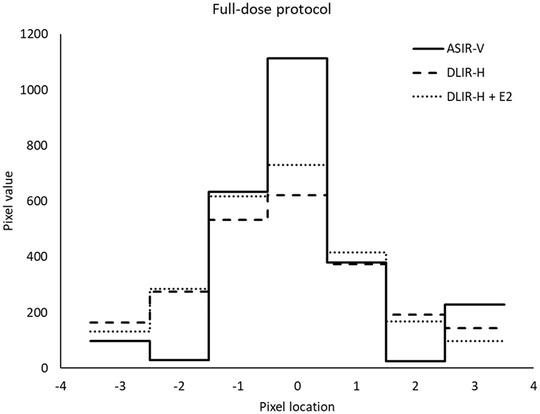
The line profiles perpendicular over a small vessel present in image slices collected using the full‐dose protocol. The solid line represents the line profile in the image reconstructed using ASIR‐V 40%, the dashed line represents the line profile in the image reconstructed using DLIR‐H and the dotted line represents the line profile in the image reconstructed using DLIR‐H + E2

**FIGURE 9:**
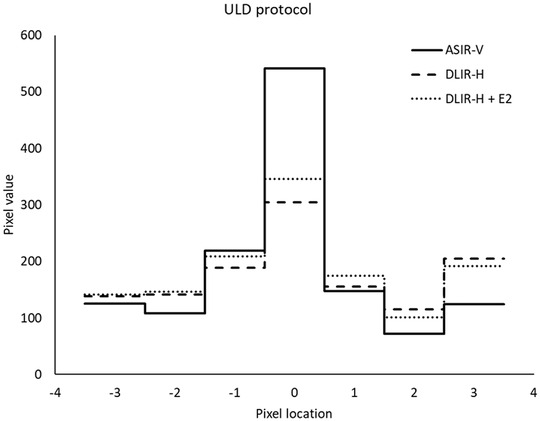
The line profiles perpendicular over a small vessel present in image slices collected using the ULD protocol. The solid line represents the line profile in the image reconstructed using ASIR‐V 40%, the dashed line represents the line profile in the image reconstructed using DLIR‐H and the dotted line represents the line profile in the image reconstructed using DLIR‐H + E2

In Figures 8 and 9 examples of line profiles perpendicular over a small vessel present in the reconstructed image slices of one of the patients included in the study are shown. Both for the full‐dose protocol and for the ULD protocol, the line profiles contain more signal in the pixel centered over the vessel (pixel location 0) for ASIR‐V 40% than for DLIR‐H and DLIR‐H + E2, indicating that the spatial resolution is slightly higher in the images reconstructed using ASIR‐V 40% than in the images reconstructed using DLIR‐H or DLIR‐H + E2. The same findings were found for line profiles plotted over small vessels present in images of other patients.

## 4 DISCUSSION

The result from the present study agrees well with the results from other studies aiming at comparing the image quality obtained from chest CT examinations reconstructed using TrueFidelity.^16^, ^17^, ^18^, ^32^ In all these studies it has been shown that TrueFidelity results in images with reduced noise, higher CNR and higher scores on subjective image quality evaluations compared to ASIR‐V. Regarding spatial resolution, the results from previous studies have shown higher low‐contrast spatial resolution in images reconstructed using DLIR than in images reconstructed using ASIR‐V, while the high‐contrast resolution was similar for ASIR‐V and DLIR. It was also shown that the spatial resolution decreases with decreasing dose level and the studies that evaluated the spatial resolution at dose levels corresponding to CTDI_vol_ ≤ 1 mGy all showed that the high‐contrast spatial resolution was lower for DLIR than for ASIR‐V at these low dose levels.

In the clinic, both the Lung kernel and the Standard kernel are often used for evaluation of chest CT examinations. As True Fidelity today is only available using the Standard kernel, it is therefore important to compare the obtained image quality using DLIR with ASIR‐V reconstructed using both the Standard kernel and the Lung kernel before introducing TrueFidelity to the clinic as a complete substitute for ASIR‐V. In the present study, the line profiles drawn perpendicular over small vessels present in patient images indicate that the spatial resolution is higher in the images reconstructed using ASIR‐V 40% (Lung kernel) than in the images reconstructed using DLIR. The same result was found for both the full‐dose protocol (mean CTDI_vol_ = 4.5 mGy) and the ULD protocol (mean CTDI_vol_ = 0.08 mGy), see examples in Figures 8 and 9. Post‐processing using an edge‐enhancement filter (DLIR‐H + E2) seems to slightly improve the spatial resolution compared to DLIR‐H, but the effect is minor. The CNR is higher in the images reconstructed using DLIR but, due to the increased noise level resulting from the edge‐enhancement filter, slightly lower in the images reconstructed using DLIR‐H + E2 than in the images reconstructed using DLIR‐H (Table 4). The image quality questions proposed by the European research project MEDIRAD (Q1–5 in the present study), were selected based on the premises that the structures are well‐defined and represent different levels of anatomical precision and underlying spatial resolution.^26^ Hence, the reproduction of these structures is theoretically benefitted by the higher spatial resolution obtained using the Lung kernel (ASIR‐V reconstruction in the present study). This is also true for Q7–9, as these image quality questions represent clinical tasks that are often evaluated using images reconstructed with a high‐resolution kernel (e.g., Lung kernel). The reproduction of lymph node 4R (Q6) and the task of diagnosing mediastinal inflammation (Q10) are mainly limited by image contrast and should therefore benefit more from the use of the DLIR (Standard kernel). This resonates with the results obtained in the present study, where Q6 and Q10 seems to benefit more from DLIR than Q1–5 and Q7–9 for both the full‐dose protocol and the ULD protocol (Figures 3 and 4). It should however be noted that the spatial resolution obtained using DLIR with Standard kernel seems to be adequate for the reproduction of the anatomical structures included in Q1–5 and for the clinical tasks represented by Q7–9, as none of these image quality questions received lower ratings for DLIR than for ASIR‐V. To conclude, despite the indicated lower spatial resolution in the images reconstructed using DLIR than in the images reconstructed using ASIR‐V 40% with Lung kernel, the results from the VGC analysis show that DLIR results in images with image quality statistically rated equal to or significantly higher than ASIR‐V 40% with Lung kernel for all evaluated image quality questions. The higher spatial resolution in images reconstructed using ASIR‐V 40% is likely out‐weighted by the improvement in image quality due to reduction in image noise that is obtained by DLIR, especially for the ULD protocol. Hence, the result of the present study indicates that there might be an opportunity to replace the two different reconstructions today commonly used for clinical evaluation of chest‐CT image with only one series reconstructed using DLIR. This could theoretically lead to reduced reading times and lower costs for image storage. However, before replacing ASIR‐V with DLIR in the clinic, additional studies comparing the detectability of pathology between ASIR‐V reconstructed using the Lung kernel and DLIR should be performed to certify that the lower spatial resolution in the DLIR images compared to images reconstructed using ASIR‐V with Lung kernel does not affect the clinical outcome of an examination.

In the present study, five observers participated in the evaluation of subjective image quality. Four of these were thoracic radiologists with more than 10 years of clinical experience and one was a radiology resident. According to Hansson et al.,^33^ it is better to handle the observers as fixed readers if the number of observers is too few and/or if the observers are not representative for the population of observers. Due to the relatively low number of observers in the present study, the fixed reader analysis was therefore chosen. However, as VGC Analyzer^29^, ^34^ provides data for both the fixed‐ and random reader situations, the effect of the results using the random reader analysis was also investigated. The random reader analysis resulted in larger confidence intervals than using the fixed reader approach. For the ULD protocol this did not affect the outcome for any of the image quality questions (DLIR‐H and DLIR‐H + E2 was still statistically rated significantly higher than ASIR‐V 40% for all questions except Q9, which was rated equal). The only difference in outcome for the ULD protocol was that no statistically significant difference between DLIR‐H and DLIR‐H + E2 was found for Q10 when the random reader approach was used. For the full‐dose protocol the statistically significant difference between DLIR‐H and ASIR‐V 40% found for Q1 and Q3 using the fixed reader analysis was no longer found using the random reader approach (DLIR‐H + E2 was however still shown to statistically be rated significantly higher than ASIR‐V 40% for Q1 and Q3). Hence, the overall the benefit of using DLIR instead of ASIR‐V 40% for full‐dose examinations seems to be a bit smaller if conclusions are drawn based on the random reader analysis.

### 4.1 Clinical usefulness of ultra‐low dose protocol

In a study by Jiang et al.^19^ nodule detection in ULD chest CT examinations (effective doses 0.07 mSv and 0.14 mSv) was evaluated, using full‐dose contrast‐enhanced CT examinations as reference. It was found that between 55.2% and 83.2% of the nodules detected in the full‐dose examinations were detected in the ULD CT examinations. The highest percentage of detected nodules was obtained in examinations performed at 0.14 mSv and reconstructed using DLIR‐H, while the lowest percentage was found for examinations performed using an exposure of 0.07 mSv reconstructed using FBP. The percentage detected nodules also varied with nodule type and size, where the highest detection rates were found for calcified nodules and nodules with a diameter ≥ 10 mm. The ULD protocol used in the present study was created in order to result in an effective dose to the patients similar to a conventional chest x‐ray examination (PA + lateral projection). Even though the images were evaluated using a slice thickness of 0.625 mm it was found that a large amount of the examinations performed using the ULD protocol were rated to have fully acceptable or probably acceptable image quality for diagnosis of pulmonary nodules (Q7, Figure 6). In fact, when TrueFidelity was used for image reconstruction only a few examinations were rated to have unacceptable image quality for this diagnostic task. An even larger number of images would probably have been rated to be fully acceptable or probably acceptable, also for diagnosis of lung emphysema and lung fibrosis, if the image evaluation would have been based on images with increased slices thickness. Hence, even if it can be argued that performing chest CT examinations at the same dose levels as conventional chest x‐ray examinations might not be clinically useful, there might still be specific clinical referrals for which the image quality obtained from ULD CT examinations might be sufficient, especially if the examinations are reconstructed using TrueFildelity. However, in the present study Q7 was interpreted by the observers as diagnosis of solid pulmonary nodules. It is known that the detection rate of nodules depends on the nodule characteristics. For example, in a study by Fletcher et al.,^35^ a reduction of radiation dose to 3–4% of the original dose level resulted in a 25% reduction in sensitivity for detection of solid pulmonary nodules with a size ≥ 5 mm in diameter, while the corresponding reduction in sensitivity was 55% for part‐solid pulmonary nodules. This fact, therefore, needs to be considered when analyzing the potential clinical benefits of ULD CT, a question that really comes to head in the discussion concerning screening for lung cancer.

In a status report on the global cancer incidence and mortality from 2018 it is shown that lung cancer is the worldwide leading cause of deaths by cancer.^36^ It has been shown that screening for lung cancer in high‐risk groups using low‐dose chest CT to a high degree reduce the mortality of lung cancer.^37^ However, the benefits of early lung cancer detection due to repeated CT examinations needs to be related to the risk of cancer induction due to the repeated exposure of ionizing radiation. Hence, the net benefit of lung cancer screening would theoretically increase if the chest CT examinations can be performed at lower exposure levels without compromising the detection rate. Even though it might not be clinically justified to use such low exposures as used for the ULD CT protocol in the present study for lung cancer screening, the results from both the present study and the study by Jiang et al.^32^ show that it might be possible to lower the exposure for chest CT examinations more than expected without extensive loss in detection rate, especially if TrueFidelity is used for image reconstruction. It must also be noted that even though examinations performed at such low dose levels as for the ULD protocol in the present study might not be valuable for detection of nodules, it might still be clinically useful if the clinical task is to follow already detected lung nodules over time.

### 4.2 Interpretation of power spectrum

In many of the previous studies aiming at evaluating the effect on noise properties from different image reconstruction techniques, the evaluation of noise properties in the images are based on calculations of noise PS in images of homogenous phantoms.^12^, ^13^, ^14^, ^15^, ^16^ Given the fact that TrueFidelity is a DLIR, the output from the reconstruction is dependent on the training data used for adjusting the different parameters included in the DNN. Hence, if the training data mostly have consisted of anatomical images, TrueFidelity might perform differently if the input data is obtained from imaging of a homogenous object instead of a patient. In the present work, the analysis of frequency components in images obtained from the different reconstruction techniques was therefore performed using patient data. Theoretically, the signal reflecting anatomy is mostly represented by relatively low frequencies and is not affected by a reduction in radiation dose. On the other hand, quantum noise is evenly distributed over the frequencies and can be expected to be higher in images collected using the ULD protocol than in the images collected using the full‐dose protocol. However, the PS curves shown in Figure 7 does not clearly reflect the theoretical assumptions. The overall differences in curve shape between ASIR‐V 40% and DLIR can partly be explained by the fact that different reconstruction kernels are used for ASIR‐V 40% (Lung kernel) and DLIR (Standard kernel). For the PS curves related to ASIR‐V 40% it is clearly seen that the ULD protocol results in images with higher noise, as the calculated PS values are higher for the ULD protocol than for the full‐dose protocol for higher frequencies. At low frequencies the signal from anatomy dominates, which explain the lack of difference in PS values between full‐dose and ULD for low frequencies. For DLIR‐H and DLIR‐ H + E2 however, the PS curves are actually higher for the full‐dose protocol than for the ULD protocol for frequencies up to approximately 0.4 mm^−1^. The reason could be that DLIR, in the ambition to lower the image noise, also reduces the signal obtained from anatomy. AI‐based image reconstruction can be seen as a non‐linear signal processing, and in all non‐linear image processing there is a risk that signal is erroneously removed. Also, if the AI‐system has not undergone extensive training based on ULD examinations, the ability of the system to distinguish between signal from anatomy and signal from noise at this low dose level might be limited, leading to the situation where signal from anatomy is erroneously removed by the system in its ambition to remove noise from the images. Additionally, if the exposure is reduced to a level where the signal from quantum noise begin to dominate over signal from anatomy also at lower frequencies, it will become increasingly difficult for the AI‐system to correctly remove noise without also remove signal from anatomy. The possibility that an AI‐based reconstruction algorithm may inadvertently reduce signal from relevant anatomy in ULD settings requires further study. However, in this study the subjective image quality evaluation results suggest that the advantage of DLIR is substantial for the ULD protocol.

Regarding the difference in noise magnitude between the images reconstructed using DLIR‐H and DLIR‐H + E2 it is clearly seen in Figure 7 that the edge‐enhancement obtained by the E2‐filter leads to a higher noise level in the images. However, the increase in noise does not seem to affect the subjective image quality. As illustrated in Figures 3 and 4, no statistically significant differences in AUC_VGC_ were obtained between examinations reconstructed using DLIR‐H and DLIR‐H + E2, except for the question related to diagnosis of mediastinal inflammation (fat stranding) (Q10) for the ULD protocol where the examinations reconstructed using DLIR‐H + E2 were statistically rated significantly higher than the images reconstructed using DLIR‐H.

### 4.3 Study limitations

One limitation of the present study is the fact that only 25 patients were included in the evaluation. The number of included patients was a trade‐off between the number of different settings for the TrueFidelity reconstructions evaluated and the image reading time for the observers. During the Delphi process only the TrueFidelity level DLIR‐H was chosen to be included in the study. It can be argued that also DLIR‐L and/or DLIR‐M should have been included in the evaluation as well. It was however obvious that all three experienced radiologists preferred DLIR‐H over DLIR‐M or DLIR‐L, why it was decided that only DLIR‐H should be included in the study. One strength of the present study is that the same patient was used both for evaluation of the full‐dose protocol and the ULD protocol. Hence, differences in benefit from using the TrueFidelity reconstruction algorithm between the two dose levels are not due to the fact that different patients were used in the evaluations.

In the present study the differences in spatial resolution between the compared reconstructions were estimated by plotting a line profile over small vessels located in the patient images. This is a quite rough estimation of the spatial resolution. There are other, more sophisticated, methods that can be used to evaluate spatial resolution in clinical CT images. For example, Sanders et al.^38^ presented a method in which the outline contour of the patient in each image slice is segmented and used to create a polygon mesh of the air‐skin boundary. The edge‐spread function (ESF) calculated across the air‐skin interface was differentiated to obtain a line‐spread function, which after Fourier transformation resulted in a CT resolution index. However, even though the method described by Sanders et al. might be a valuable tool to perform a more thorough evaluation of the spatial resolution in clinical CT images, one must be aware that the nonlinearity of reconstructed CT images may limit the validity of the method and make the interpretation of the results difficult.

## 5 CONCLUSION

In the present study examinations reconstructed using TrueFidelity (DLIR‐H or DLIR‐H + E2) were statistically rated equal to (AUC = 0.5) or significantly higher (AUC > 0.5) than examinations reconstructed using ASIR‐V 40% for all image quality questions. The benefit of using TrueFidelity instead of ASIR‐V 40% for image reconstruction seems to be higher for the ULD protocol than for the full‐dose protocol. Post‐processing of the images reconstructed using TrueFidelity using an edge‐enhancement filter (DLIR‐H + E2) was shown to increase the noise magnitude compared to images obtained using TrueFidelity reconstruction without post‐processing (DLIR‐H). However, the increase in noise did not affect the image quality ratings and in general no statistically significant differences could be found between DLIR‐H and DLIR‐H + E2.

## AUTHOR CONTRIBUTIONS

Angelica Svalkvist: Main responsibility for study design, study setup, and analysis of results. Main responsibility for drafting the manuscript. Have approved to the final version to be published. Erika Fagman: Substantial contribution to study design, study setup and interpretation and discussion of the obtained results. Have critically reviewed the manuscript and approved to the final version to be published. Jenny Vikgren: Substantial contribution to study design and interpretation and discussion of the obtained results. Have critically reviewed the manuscript and approved to the final version to be published. Sara Ku: Substantial contribution to patient recruitment, image acquisition, and study setup. Have critically reviewed the manuscript and approved to the final version to be published. Micael Oliveira Diniz: Substantial contribution to interpretation and discussion of the obtained results. Have critically reviewed the manuscript and approved to the final version to be published. Rauni Rossi Norrlund: Substantial contribution to interpretation and discussion of the obtained results. Have critically reviewed the manuscript and approved to the final version to be published. Åse A. Johnsson: Substantial contribution to study design, study setup and interpretation and discussion of the obtained results. Have critically reviewed the manuscript and approved to the final version to be published.

## CONFLICT OF INTEREST

No conflicts of interest.

## ETHICS STATEMENT

The Swedish Ethical Review Authority approved this study (reference number 2020‐00581), and all participants gave written informed consent.
